# Supplementary material for: The Identification and Gene Mapping of Spotted Leaf Mutant spl43 in Rice
Source: Int J Mol Sci. 2024 Jun 17;25(12):6637. doi: 10.3390/ijms25126637 (PMC11203680; doi:10.3390/ijms25126637)
Supplement: Supplementary file 1 [file ijms-25-06637-s001.zip › ijms-3011203-supplementary.pdf]

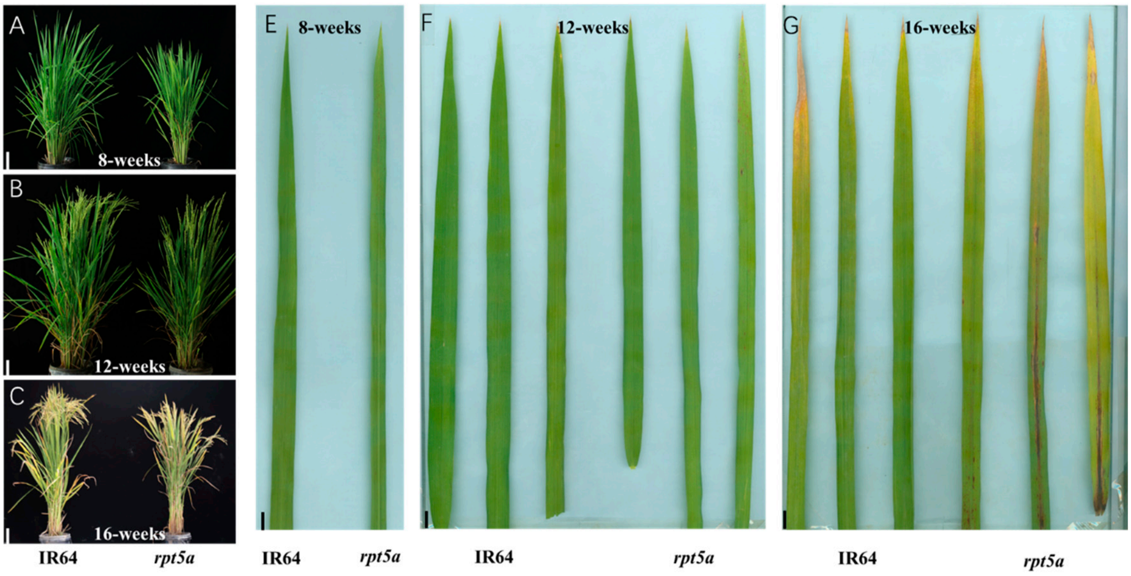

Supplemental Figure S1 The phenotypes of IR64 and *rpt5a*.

(A): Phenotypes of IR64 and *rpt5a* at 8 weeks, 12 weeks and 16 weeks. Bar = 20 cm; (B): Leaf phenotypes of IR64 and *rpt5a* at 8 weeks, 12 weeks and 16 weeks. Bar = 1 cm;

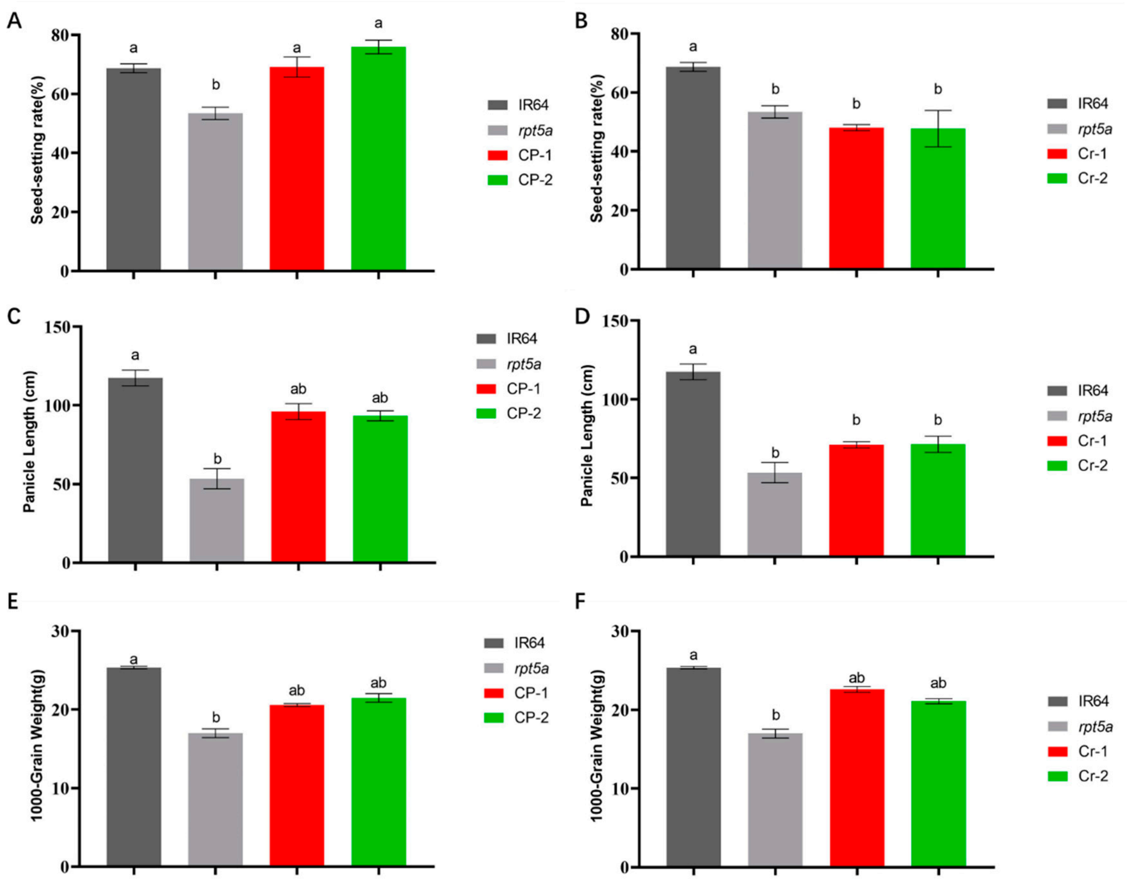

Supplemental Figure S2 Agronomic traits of IR64, *rpt5a* and complementary lines (CP-1, CP-2), knockout lines (Cr-1, Cr-2).

(A, B): Seed-setting rate; (C, D): Panicle length; (E, F): 1000-Grain weight. Different letters signify statistically significant differences, as assessed by one-way ANOVA followed by Duncan's multiple range test, with a significance threshold of $P \leq 0.05$; Error bars represent SD (n = 3).

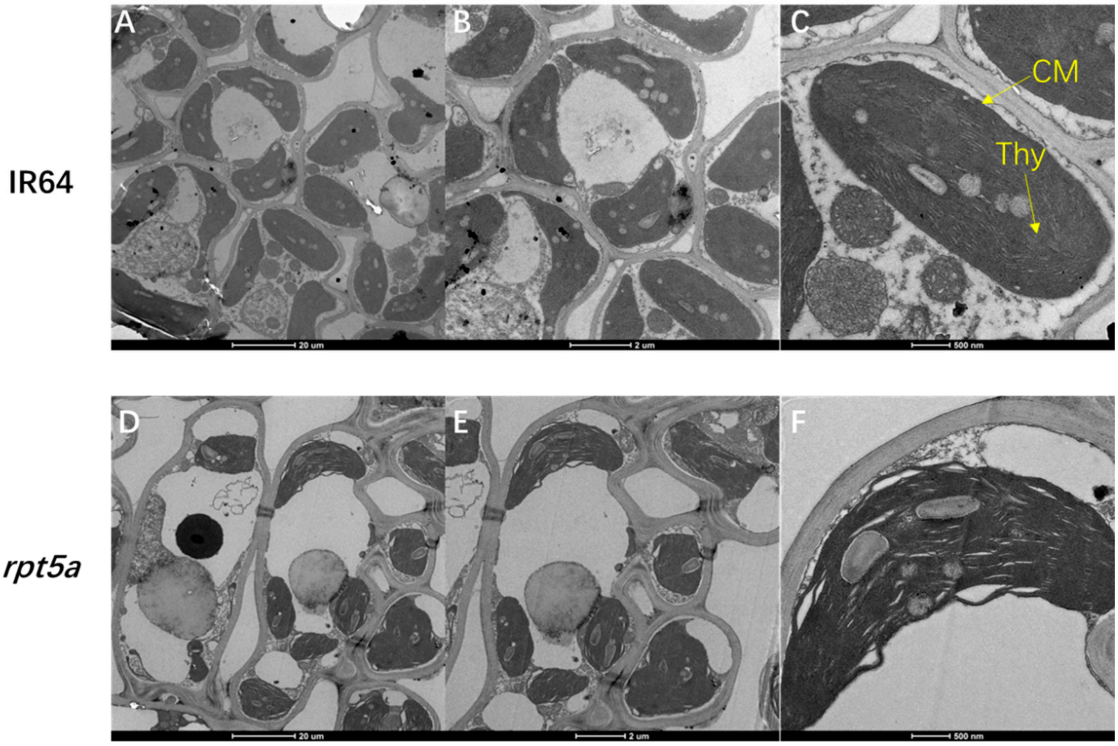

Supplemental Figure S3 TEM observation of the chloroplasts

(A, B, C): TEM observation of the chloroplast in IR64 at the tillering stage; (D, E, F): TEM observation of the chloroplast in *rpt5a* at the tillering stage. Thy, thylakoid lamellae; CM, chloroplast membrane.

Table S1 Primers used in this study

Name	Primer (5'-3')	Purpose
RM6-F	GTCCCCTCCACCCAATTC	Gene mapping
RM6-R	TCGTCTACTGTTGGCTGCAC	Gene mapping
RM250-F	GGTTCAAACCAAGCTGATCA	Gene mapping
RM250-R	GATGAAGGCCTTCCACGCAG	Gene mapping
RM14229-F	CCCAACCTTAATTCCTCCATCTGC	Gene mapping
RM14229-R	CCCATGGCCTTCCAAATTAAGG	Gene mapping
D237D-F	GGTCGGTCGAGACGTGTAGA	Gene mapping
D237D-R	TTCCCAGAGCACCTTTCATA	Gene mapping
D240A-F	TCTGTCAGTTCTGGCATTGTT	Gene mapping
D240A-R	GAGATTTCACGGGCTTTGTAT	Gene mapping
D240B-F	TGATCTCTCTCCGTAATTTACTG	Gene mapping
D240B-R	ATATGGTTTATGTGGACGCTATA	Gene mapping
C154-2-F	CGAGATACTCCCTCCGTATCA	Gene mapping
C154-2-R	AGCCTAGTTACCTTGGTCCT	Gene mapping
C154-11-F	GCCCTTCACCAATCATTGTA	Gene mapping
C154-11-R	TTGTCTAAGGAACTCAGATCACG	Gene mapping
C154-27-F	AGCAAACTCACTTCCAATAGCA	Gene mapping
C154-27-R	CAGCAGCATTGGCATGTTTA	Gene mapping
PCAMBIA1300-RPT5A-F	tatgaccatgattacgaattc<br>AGCTAGCACTGGAACAACATCT	Complementation
PCAMBIA1300-RPT5A-R	acgacggccagtgccaagctt<br>GCCCCAAAGTTAAGCGACAC	Complementation
CR-Target-F	GGCAGATAGCTGCAACTAATCGCG	Knockout vector construction
CR-Target-R	AAACCGCGATTAGTTGCAGCTATC	Knockout vector construction
pCambia1381Z-GUS-F	tatgaccatgattacgaattc<br>AACGCAATTAATGTGAGTTAGCTCA	GUS assay
pCambia1381Z-GUS-R	tggctgcaggtcgacggatcc<br>CGCTAGGGATTTTGCTTCGA	GUS assay
GFP-RPT5A /RPT5^{AV318E}-F	cgctctagaactagtggatcc<br>ATGTCGTCGCCGCCGCCC	Subcellular localization
GFP-RPT5A /RPT5^{AV318E}-R	gataagcttgatatcgaattc<br>AGCGTAATAATTTAAACTGGACTTCTTT	Subcellular localization
